# *Klebsiella pneumoniae* Chorioamnionitis: An Underrecognized Cause of Preterm Premature Rupture of Membranes in the Second Trimester

**DOI:** 10.3390/microorganisms9010096

**Published:** 2021-01-03

**Authors:** Maria Paola Bonasoni, Andrea Palicelli, Giulia Dalla Dea, Giuseppina Comitini, Paola Nardini, Loredana Vizzini, Giuseppe Russello, Marcellino Bardaro, Edoardo Carretto

**Affiliations:** 1 Pathology Unit, Azienda Unità Sanitaria Locale-IRCCS di Reggio Emilia, 42122 Reggio Emilia, Italy; Andrea.Palicelli@ausl.re.it; 2 Pathology Unit, “Maggiore della Carità” Hospital, 28100 Novara, Italy; gdalladea12@gmail.com; 3 Department of Obstetrics & Gynaecology, Azienda Unità Sanitaria Locale-IRCCS di Reggio Emilia, 42122 Reggio Emilia, Italy; Giuseppina.Comitini@ausl.re.it; 4 Clinical Microbiology Laboratory, IRCCS Arcispedale Santa Maria Nuova, 42122 Reggio Emilia, Italy; Paola.Nardini@ausl.re.it (P.N.); Loredana.Vizzini@ausl.re.it (L.V.); Giuseppe.Russello@ausl.re.it (G.R.); Marcellino.Bardaro@ausl.re.it (M.B.); Edoardo.Carretto@ausl.re.it (E.C.)

**Keywords:** *Klebsiella pneumoniae*, preterm premature rupture of membrane, chorioamnionitis

## Abstract

*Klebsiella pneumoniae* is a Gram-negative, rod-shaped bacterium, responsible for hospital and community acquired pneumonia, urinary tract and wound infections, and bloodstream dissemination. *K. pneumoniae* infection in pregnancy, leading to acute chorioamnionitis (AC), preterm premature rupture of membranes (PPROM) and early pregnancy loss in the second trimester, has been rarely reported. Herein, we present a case of *K. pneumoniae* AC that caused intrauterine fetal demise (IUFD) at 19 weeks + 5 days. The 36-year-old mother was admitted at 18 weeks + 1 day of gestation for threatened abortion. IUFD occurred 11 days after. Fetal postmortem showed severe AC and funisitis, neutrophils within alveoli and intestinal lumen, associated with rod-like bacteria. Fetal blood and lung cultures grew *K. pneumoniae*, β-lactamase-non-producing strain. Antibiogram revealed sensitivity for piperacillin/tazobactam. Three days after IUFD, the mother presented with fever (37.8 °C) which persisted for one week. Maternal blood and urine cultures were negative. According to fetal microbiological results, available 6 days after IUFD, initial treatment with amoxicillin/clavulanic acid was replaced with piperacillin/tazobactam with full patient recovery. Therefore, in the event of PPROM and IUFD, fetal microbiological investigations should always be performed to isolate the proper etiologic agent and start the correct medical treatment.

## 1. Introduction

Acute chorioamnionitis (AC) is defined as infiltration of neutrophils within placental chorionic plate and amniochorial membranes, usually due to ascending infection [1,2]. Although AC often occurs as subclinical condition, diagnosed only by placental histological examination [3], bacterial infection of the amniotic cavity is the main cause of preterm premature rupture of membranes (PPROM) leading to preterm delivery, respiratory distress, sepsis, and occasionally fetal/neonatal death [4,5,6,7,8]. Moreover, AC is a high risk factor for neonatal necrotising enterocolitis (NEC), retinopathy of prematurity (ROP), poor long-term neurologic outcome, and cerebral palsy [4,5,6,7,8]. The main microorganisms responsible for AC are group B *Streptococcus*, *Fusobacterium nucleatum*, *Peptostreptococcus*, *Escherichia coli*, *Bacteroides* species, *Ureaplasma urealyticum*, and *Listeria monocytogenes* [1,2]. The gold standard for AC diagnosis remains placental examination. [6]. However, microorganism identification, and subsequent antibiogram, relies on fetal blood and tissue cultures, placental subchorionic fibrin swab, and parenchymal cultures [9,10,11].

To date, intrauterine infection and fetal demise due to *K. pneumoniae* has been described in only three cases. All of them were second trimester pregnancies presented with PPROM and early pregnancy loss [12,13,14].

Herein, we report a new case of AC due to *K. pneumoniae* leading to fetal death at 19 weeks + 5 days of gestation. Infection was confirmed by fetal blood and tissue microbiological cultures. We also discuss maternal clinical presentation and antibiotic treatment.

## 2. Case Description

### 2.1. Mother Clinical Presentation and Treatment

A 36-year-old woman was admitted to our Institution for threatened abortion at 18 weeks + 1 day of gestation (wga) and treated with antibiotics according to international PPROM guidelines [15]. The treatment consisted of ampicillin and azithromycin for 2 days, then replaced with amoxicillin/clavulanic acid for 5 days. The patient was carefully monitored, but at day 11 after hospital admission, transabdominal ultrasound (US) revealed intrauterine fetal death (IUFD) associated with oligohydramnios. Labor was induced and a male stillborn was vaginally delivered after 36 h.

At day 12 after admission, the patient was treated with ampicillin and gentamicin.

At day 14, the patient presented with fever (37.8 °C) and elevated C-reactive protein (CRP) of 11.10 mg/dL, however blood and urine culture were negative.

At day 16, due to fever persistence and increased CRP of 17.56 mg/dL, antibiotic therapy was modified with amoxicillin/clavulanic acid.

At day 18, as the patient was still feverish with high CRP (12.06 mg/dL), the previous therapy was suspended and replaced with piperacillin/tazobactam.

The patient fully recovered with fever remission and CRP reduction, and after 3 days without fever, she was discharged (day 21).

### 2.2. Fetal Autopsy and Microbiological Results

IUFD was diagnosed 11 days after the patient’s admission, corresponding approximately to a gestational age of 19 weeks + 5 days.

Postmortem examination revealed a nonmacerated fetus weighing 240 g and measuring 24.5 cm in crown–heel length. The other measurements were as follows: crown–rump length 17.5 cm; foot length 3 cm; head, chest, and abdominal circumference 16 cm, 13 cm, and 12.5 cm, respectively. Overall, anthropometric measurements were consistent with 19 weeks’ gestation [16]. External examination showed a normal male fetus with mild facial and nuchal oedema. Internal examination disclosed minimal pleural and abdominal serous effusions and organ congestion. No congenital anomalies were found.

**Figure 1:**
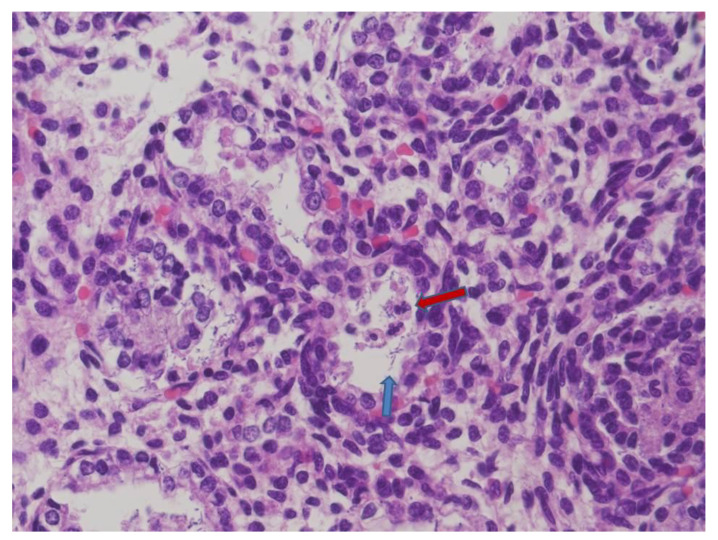
Fetal lung: a few intra-alveolar neutrophils (red arrow) associated with rod-shaped bacteria (blue arrow) (HE staining 20×).

**Figure 2:**
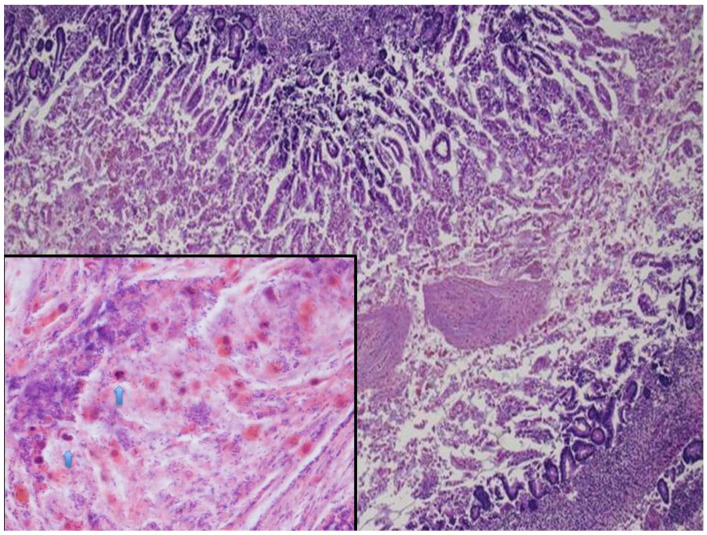
Fetal small intestine: intestinal lumen filled with mucus (HE staining 4×). In the mucoid material (frame) there were neutrophils (blue arrows) intermixed with abundant clusters of rod-shaped bacteria (HE staining 20×).

**Figure 3:**
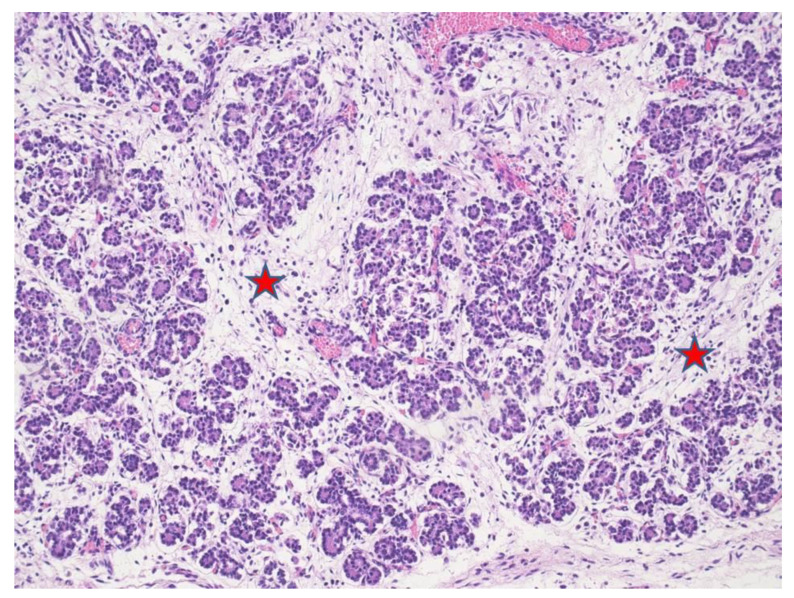
Pancreatic acini with interacinar edema (red stars) (HE staining 4×).

At microscopy, lungs displayed mild pneumonia with focal intra-alveolar neutrophils associated with rod-like bacteria (Figure 1). Neutrophils and rod-like bacteria were similarly observed in the lumen of the gastrointestinal tract (Figure 2). No other significant histological findings were noted except for mild pancreatic oedema (Figure 3).

Postmortem lung and blood cultures were processed according to routine procedures on selective media. Identification was performed by MALDI-ToF MS (MALDI Biotyper, Bruker Daltonik GmbH, D-28359, Bremen, Germany), while for susceptibility testing, the Phoenix 100™ system was used (Becton, Dickinson and Company, Franklin Lakes, NJ, USA) and agar diffusion (Kirby–Bauer method, according to EUCAST rules).

The isolate was identified as *K. pneumoniae* ssp. *pneumoniae*. Further phenotypical test, performed according to Brisse et al., allowed for setting the isolate in the *K. pneumoniae* phylogroup KpI [17].

The isolate’s susceptibility profile revealed a wild-type phenotype, being susceptible to all the antibiotics tested, except for the intrinsic resistances (Intrinsic_Resistance_and_Unusual_Phenotypes_Tables_v3.2_20200225 in https://www.eucast.org/expert_rules_and_intrinsic_resistance). No ESBL, AmpC, or carbapenemase resistance traits were phenotypically documented, according to EUCAST rules EUCAST_detection_of_resistance_mechanisms_170711, in https://www.eucast.org/resistance_mechanisms).

On blood agar, the isolate did not show the hypermucoviscosity (HMV) phenomenon, as demonstrated by the negativity on the string test, where a standard bacteriological loop was used to stretch a mucoviscous string from the bacterial colony [18].

The antibiogram revealed sensitivity for amoxicillin/clavulanic acid, gentamicin, piperacillin/tazobactam; and resistance to ampicillin (Table 1).

Microbiological results approximately arrived after 17 days of patient’s hospital admission and communicated to the clinicians. As the patient was still feverish with elevated CRP, the treatment was changed accordingly from amoxicillin/clavulanic acid to piperacillin/tazobactam.

**Figure 4:**
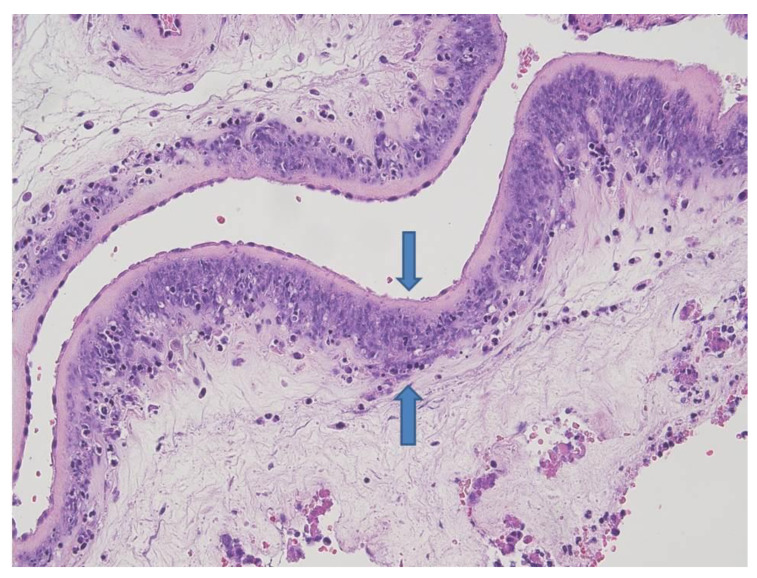
Amniochorial membranes: severe acute chorioamnionitis with focal amnion necrosis (between blue arrows) (HE staining 10×).

**Figure 5:**
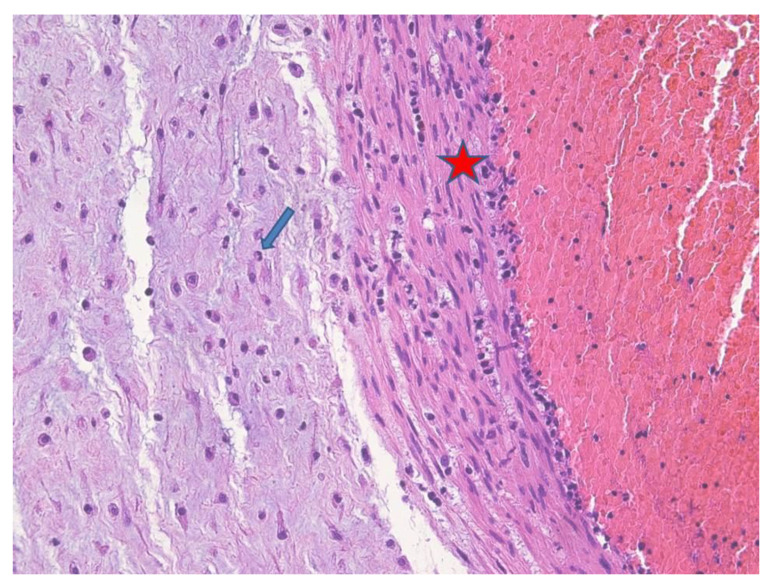
Umbilical vein acute flebitis: the umbilical vein showed acute parietal inflammation (red star) with extension to the Wharton’s jelly (blue arrow) (HE staining 20×).

The placenta was received fragmented and weighed 139 g. Macroscopically, recognizable strands of membranes were yellowish and opaque. Microscopically, there was severe chorioamnionitis with focal amnion necrosis (Figure 4) corresponding to a maternal inflammatory response stage 3/3 and grade 2/2 [19]. Bacterial organisms were noted at high magnification. Funisitis was also observed with neutrophilic infiltrate of the umbilical vein, one artery, and extension to Wharton’s jelly (Figure 5). These findings were consistent with fetal inflammatory response stage 2/3 and grade 2/2 [19].

The placental parenchyma was normally developed for second trimester, with mesenchymal and immature intermediate villi. Multifocal villous oedema was also identified. The decidua showed diffuse acute necrotizing deciduitis and focal laminar necrosis.

## 3. Materials and Methods

We searched for ((chorioamnionitis OR ((placenta OR placental) AND (infection OR inflammation)) OR PROM OR “premature rupture of membranes”) AND *K. pneumoniae* in Pubmed (all fields, 55 results), Scopus (Title/Abstract/Keywords, 15 results), and Web of Science (Topic/Title, 25 results) databases. No limitations were set. Titles and abstract of the results were screened to identify relevant articles. All relevant papers were obtained in full-text format and screened for additional references. The bibliographic research ended on 1 October 2020: three papers were finally included.

## 4. Discussion

*K. pneumoniae* is a Gram-negative, rod-shaped bacterium belonging to the family of *Enterobacteriaceae*, involved in hospital and community-acquired bacterial pneumonia, urinary tract, and wound infections [20,21].

*K. pneumoniae* represents the main cause of hospital-acquired infections (HAI), and as an opportunistic pathogen, typically induces infections in hospitalized or immunocompromised patients.

Behind *Clostridium difficile* and *Staphylococcus aureus*, *K. pneumoniae* is the third main pathogen responsible for HAI, defined as the onset of pneumonia in ≥48 h after hospital admission [22]. Mortality rate in *K. pneumoniae* pneumonia is high, reaching 50% [20].

Community-acquired *K. pneumoniae* pneumonia is usually severe, panlobar, and often diagnosed in chronic alcoholics, attributed to aspiration of gastric contents. This kind of pneumonia is also known as ‘‘Friedlander’s pneumonia’’ characterized by radiographic alterations due to severe pyogenic infection [23]. Urinary tract is often infected by *K. pneumoniae*, especially in patients with diabetes mellitus or neuropathic bladder, mainly occurring in a nosocomial setting [20,21]. A typical complication is catheter-associated urinary tract infections (CAUTIs) due to the bacterium ability for building biofilms and adhering to catheters [24].

*K. pneumoniae* may also colonize wound/surgical sites, representing almost 13% of all the infections caused.

*K. pneumoniae* is the second cause of bloodstream infections (BSI), triggered by Gram-negative bacteria, behind only to *Escherichia coli* [20,21,22].

BSI may be hospital acquired or occurring in a community setting. In the first situation, cancer is the main underlying disease. Instead, diabetes mellitus and chronic hepatic conditions are typically found in the community [25]. Bacteremia is usually caused by a secondary dissemination from a known site of infection. Typical sources include the urinary and gastrointestinal tract, intravenous or urinary catheters, and respiratory localization [26].

In the neonatal population, *K. pneumoniae* infection can be responsible for neonatal sepsis and meningitis and may affect premature infants and spread within pediatric wards [20,27,28].

However, to the best of our knowledge, intrauterine fetal death due to *K. pneumoniae* infection has been reported only in three cases [12,13,14].

Sheikh et al. [12] described a case of IUFD at 18 weeks of gestation. AC and acute villitis were found in the placenta. *K. pneumoniae* was isolated in maternal blood and placental cultures. Fetal autopsy was not performed. The mother was admitted to the hospital with high temperature (41 °C) and malodorous vaginal discharge. The patient had also vaginal bleeding since one day before the admission.

The PPROM presented by Omwandho et al. [13] was a spontaneous miscarriage at 15 weeks. The mother had a threatened miscarriage at 12th week of gestation with light vaginal bleeding, but then she completely recovered after staying in bed. However, at 15th week, US showed IUFD. Placental cultures were positive for *K. pneumoniae*, but no pathology was seen in the placenta. Fetal autopsy was also negative for infection or inflammation. Nevertheless, the authors attributed the fetal demise to *K. pneumoniae* placental microbiological finding. The husband suffered from *K. pneumoniae* prostatitis and likely infected his wife.

Torabi et al. reported [14] PPROM and IUFD at 20 weeks of gestation due to *K. pneumoniae* intrauterine infection as the bacterium was found in fetal blood and lung tissue cultures. Placental pathology showed severe AC, chorionic vasculitis, and funisitis. Fetal autopsy showed clusters of neutrophils and bacteria both in the lungs and in the lumen of the gastrointestinal tract. The patient was admitted to the hospital with PPROM, but she had no vaginal bleeding before, and no fever prior to or during the admission. She also denied respiratory or urinary infections for the duration of her pregnancy.

In our case, the patient was admitted for threatened abortion at 18 wga +1 day with no fever. Although she underwent antibiotic treatment (ampicillin and azithromycin for 2 days, and then amoxicillin/clavulanic acid for 5 days), IUFD was diagnosed at 19 wga + 5 days. Ampicillin and gentamicin were started. Three days after IUFD, the patient was feverish (37.8 °C) with high CRP. Fever and elevated CRP persisted for another 7 days. However, maternal blood and urine cultures were negative. First, antibiotic therapy was modified with amoxicillin/clavulanic acid with no results. Then, after 6 days since IUFD, postmortem fetal lung and blood cultures identified *K. pneumoniae*, β-lactamase-non-producing strain. The antibiogram showed sensitivity for amoxicillin/clavulanic acid, gentamicin, piperacillin/tazobactam; and resistance to ampicillin. Then, accordingly to these findings and 7 days after IUFD, medical treatment was replaced with piperacillin/tazobactam with complete symptom resolution and CRP reduction.

Similarly to Torabi’s case [14], placental examination showed severe AC and funisitis. Neutrophils and rod-shaped bacteria were found in fetal lungs and in the lumen of the gastrointestinal tract.

In the management of our case, microbiological cultures on fetal blood and lung tissue, including the antibiogram, were of paramount importance in identifying *K. pneumoniae* as the etiologic agent, and mother’s medical treatment was then changed accordingly.

However, to date, PPROM and IUFD due to *K. pneumoniae* have been scarcely reported. One main reason may be attributed to the lack of submitting fetal tissues for microbiological studies.

Although microbiological studies are always recommended in case of PPROM as indicated in the perinatal autopsy protocol, in common practice only fetal autopsies carried out by dedicated perinatal pathologists are performed correctly, including ancillary studies [9].

Another cause may be failure in microbiological culture, as bacteria could not grow or they may be difficult to cultivate [29].

It must be taken into account that AC may be clinically silent and the hallmark for diagnosis relies on placental histological examination [30]. In our specific case, AC and fetal infection were observed, including rod-shaped bacteria. Microbiological cultures on postmortem fetal blood and tissues, and subsequent antibiogram, grew *K. pneumoniae* and provided the correct maternal treatment.

In the case we described, *K. pneumoniae*, acquired as ascending infection, determined AC. However, bacterial specific source remained undetermined. The mother denied previous urinary or respiratory infections. In humans, *K. pneumoniae* is a saprophyte in the nasopharynx and in the intestinal tract. Carrier rates are variable, but isolation from stools is usually higher [22]. Carrier rates typically worsen in a hospital environment. Reported carrier rates in hospitalized patients are 77% in the stool, 19% in the pharynx, and 42% on the hands of patients. The high rates detected in hospital settings are usually associated with antibiotic therapy and progressively increase with the length of stay [20]. It is only a speculation that in our case, AC might have occurred during the hospital stay as a consequence of carrier exacerbation.

Therefore, in the event of PPROM leading to a miscarriage, microbiological cultures on fetal blood and tissues should be mandatory, in order to find the right etiologic agent, and consequent antibiotic therapy.

By and large, appropriate management of clinical chorioamnionitis has been recently reviewed by a workshop of experts [31,32]. The effort has been made to improve maternal and fetal/neonatal wellbeing in order to reduce overall morbidity and unnecessary antibiotic treatment.

First, the term “chorioamnionitis”, in a clinical setting, should be avoided and restricted to the histopathological diagnosis of amniochorial membrane inflammation and/or funisitis. Instead, the new concept of “Triple I” is introduced to indicate “Intrauterine Inflammation or Infection or both”.

Triple I is diagnosed when there is maternal fever with one or more of the following: (1) fetal tachycardia (>160 bpm for 10 min or longer); (2) maternal WBC >15,000 in absence of corticosteroid; (3) purulent fluid from the cervical or confirmed visually on speculum; (4) biochemical or microbiologic amniotic fluid (AF) consistent with amniotic infection.

Triple I should be further classified as “suspected” or “confirmed”. Triple I confirmation requires evidence of infection either in AF (positive Gram stain for bacteria, low AF glucose, high WBC count in the absence of a bloody tap, or positive AF culture results) or in placental histopathological examination (e.g., chorioamnionitis and/or funisitis). Without the previous criteria, Triple I remains as “suspected” or “isolated maternal fever”, the latter categorized as “not Triple I”. There is still controversy regarding reliable antenatal and postnatal biomarkers able to accurately detect the neonatal risk for early onset sepsis (EOS). For example, prenatal dosage of IL-6 value seems promising in assessing the severity of intrauterine infection. The hallmark for EOS still relies on neonatal blood cultures, however they can be biased by false-negative or false-positive results. Moreover, a close communication has been recommended between the obstetric and neonatal team about maternal and fetal/neonatal conditions, e.g., confirmation of Triple I, maternal antibiotic or antipyretic treatment, to name a few.

Triple I may have potential complications such as postpartum hemorrhage, wound infection, and endomyometritis [33]. Antimicrobial treatment depends on Triple I features; however, a wide spectrum therapy with ampicillin and gentamicin is usually given. In case of cesarean section, clindamycin or metronidazole should be added to cover anaerobic pathogens. Bacteremia, sepsis, and persistent fever will be taken into account to determine the duration of treatment. Newborns at risk of EOS tend to receive antibiotics for almost 5 days; conversely it is still debated in wellbeing infants if the treatment is necessary for more than 48 h.

However, a close communication between the obstetric and neonatal teams is paramount for a correct management of maternal and fetal/neonatal health care. In this context, placental examination plays a key role either in identifying the microorganism through tissue cultures or histologically confirming a Triple I [31,32].

Consequently, a good interaction between pathologists and clinicians must also be put in place, as happened in the case we described, in which isolation of *K. pneumoniae* in fetal blood and tissues, helped to change maternal antibiotic treatment.

## Figures and Tables

**Table 1:** *Klebsiella pneumoniae* antibiogram. MIC: minimum inhibitory concentration.

Antibiotic	MIC µg/mL	Resistance/Susceptibility
amikacin	<=4	S
amoxicillin/clavulanic acid	<4/4	S
ampicillin	>8	R
aztreonam	<=1	S
cefepime	<=1	S
ceftazidime	<=0.5	S
ceftriaxone	<=0.5	S
ciprofloxacin	<=0.25	S
colistin	0.5	S
ertapenem	<=0.25	S
fosfomycin	32	S
gentamicin	<=1	S
imipenem	0.5	S
levofloxacin	<=0.5	S
meropenem	<=0.125	S
piperacillin/tazobactam	<4/4	S
tobramicyn	<=1	S
trimethoprim/sulfamethoxazole	<=1/19	S
